# Einstein-Podolsky-Rosen Steering Inequalities and Applications

**DOI:** 10.3390/e20090683

**Published:** 2018-09-07

**Authors:** Ying Yang, Huaixin Cao

**Affiliations:** 1 School of Mathematics and Information Science, Shaanxi Normal University, Xi’an 710062, China; 2 School of Mathematics and Information Technology, Yuncheng University, Yuncheng 044000, China

**Keywords:** steerability, unsteerability, steering inequality

## Abstract

Einstein-Podolsky-Rosen (EPR) steering is very important quantum correlation of a composite quantum system. It is an intermediate type of nonlocal correlation between entanglement and Bell nonlocality. In this paper, based on introducing definitions and characterizations of EPR steering, some EPR steering inequalities are derived. With these inequalities, the steerability of the maximally entangled state is checked and some conditions for the steerability of the X-states are obtained.

## 1. Introduction

Generally, quantum correlations means the correlations between subsystems of a composite quantum system, including Bell nonlocality, steerability, entanglement and quantum discord.

Einstein-Podolsky-Rosen (EPR) steering was first observed by Schrodinger [1] in the context of famous Einstein-Podolsky-Rosen (EPR) paradox [2,3,4,5]. It was realized that EPR steering, as a form of bipartite quantum correlation, is an intermediate between entanglement and Bell nonlocality. Wiseman et al. [6] shown the inequivalence between entanglement, steering, and nonlocality when considering the projective measurements. Then, Quintino et al. [7] further considered the general measurements and proved that these three quantum relations are inequivalent. Interestingly, steering can be characterized by a simple quantum information processing task, namely, entanglement verification with an untrusted party [6,7,8,9,10]. In addition, steering has been found useful in several applications, such as one-sided device-independent quantum key distribution [11]; subchannel discrimination [12]; temporal steering and security of quantum key distribution with mutually unbiased bases against individual attacks [13]; temporal steering in four dimensions with applications to coupled qubits and magnetoreception [14]; no-cloning of quantum steering [15]; and spatio-temporal steering for testing nonclassical correlations in quantum networks [16]. Recently, detection and characterization of steering have attracted increasing attention [3,6,8,17,18,19,20,21,22,23,24,25,26,27,28,29,30,31,32]. Many of the standard Bell inequalities (e.g., CHSH) are not effective for detection of quantum correlations which allow for steering, because for a wide range of such correlations they are not violated. Various steering inequalities have been derived, such as linear steering inequalities [33,34,35]; inequalities based on multiplicative variances [3,17,33]; entropy uncertainty relations [36,37]; fine-grained uncertainty relations [38], temporal steering inequality [39]. Besides, Zukowski et al. [40] presented some Bell-like inequalities which have lower bounds for non-steering correlations than for local causal models. These inequalities involve all possible measurement settings at each side. Based on the data-processing inequality for an extended Rényi relative entropy, Zhu et al. [41] introduced a family of steering inequalities, which detect steering much more efficiently than those inequalities known before. Chen et al. [42] showed that Bell nonlocal states can be constructed from some steerable states. Furthermore, a nine-setting steering inequality had also been presented for developing more efficient one-way steering and detecting some Bell nonlocal states. Bhattacharya et al. [43] present absolute non-violation of a three-setting steering inequality by two-qubit states. Recently, some characterizations of EPR steering are given in [44] and the generalized steering robustness was introduced and some interesting properties were established in [45], which suggests a way of quantifying quantum steering. Very recently, Bell nonlocality and EPR steering of bipartite states were discussed mathematically in [46], including mathematical definitions and characterizations of these two quantum correlations, the convexity and closedness of the sets of all Bell local states and all EPR unsteerable states, respectively. Lastly, a sufficient condition for a state to be steerable was established, which leads to proofs of the EPR steerability of the maximally entangled states and that of entangled pure states. Tripartite systems have more complex structures than bipartite systems and then have more diversified steering scenarios. In [47], two types of quantum steering scenarios were introduced for a tripartite quantum system, named “one-sided device-independent steering”and “two-sided device-independent steering”. Based on giving the mathematical definitions of these steering scenarios, some necessary and sufficient conditions for a state to be unsteerable were obtained and sufficient conditions for a state to be steerable were established.

In this paper, we will derive some EPR steering inequalities for bipartite states, including a more general steering inequality that extends some known steering inequalities. Furthermore, we derive some EPR steering criteria, with which the EPR steerability of the maximally entangled states and Bell-diagonal states are checked. The other parts of this paper are divided as follows. In Section 2, we will introduce the definitions of EPR unsteerability and EPR steerability of bipartite states, and some equivalent characterizations of EPR unsteerability. In Section 3, we will establish some EPR steering inequalities, prove the steerability of the maximally entangled state and derive some conditions for the steerability of the X-states.

## 2. Steering Inequalities of Bipartite Quantum States

In this section, we will recall mathematical definitions related to steering motivated by the literature (e.g., [29]) and proposed in [46], and list related results proved in [46]. To do this, we use $H_{A}$ and $H_{B}$ to denote two finite dimensional complex Hilbert spaces, which describe two quantum systems *A* and *B*, respectively. We use $D_{X}$ to denote the set $D(H_{X})$ of all quantum states of the system *X* described by a Hilbert space $H_{X}$ and $1_{X}$ to denote the identity operator on $H_{X}$.

**Figure 1:**
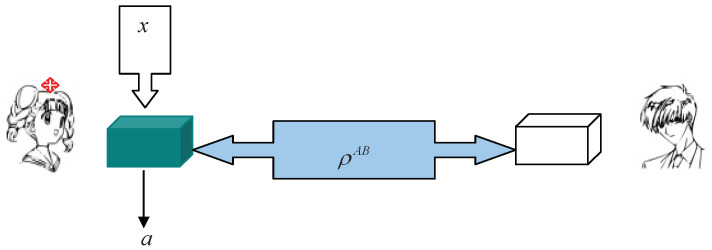
Sketch of a quantum steering from Alice to Bob, in which $\rho^{AB}$ denotes the shared state and *x* and *a* denote Alice’s measurement choice and corresponding outcome, respectively, when the measurement *x* is chosen and performed.

In a typical quantum-steering scenario, there are two spatially separated systems *A* and *B*, which are measured by one of the two distant observers, Alice and Bob; they share a joint state $\rho^{AB}$ (Figure 1). Alice may choose one measurement, labeled by *x*, from her measurement assemblage $M_{A}$, and perform it on her system *A*. Bob performs tomography and reconstructs the set of states

$$\rho_{a|x}={\mathrm{tr}}_{A}\left[\left(M_{a|x}⊗I_{B}\right)\rho^{AB}\right]$$

conditioned on Alice’s measurements. The aim of this experiment is to steer Bob’s state using Alice’s measurement on her system.

Here are the mathematical definitions concerning EPR steering given by [46].

**Definition** **1.** *Let $M_{A}=\left\{{\left\{M_{a|x}\right\}}_{a=1}^{o_{A}}:x=1,2,\ldots ,m_{A}\right\}$ be a set of $m_{A}$ positive operator value measurements (POVMs) ${\left\{M_{a|x}\right\}}_{a=1}^{o_{A}}\left(x=1,2,\ldots ,m_{A}\right)$ that Alice want to perform, called a measurement assemblage of Alice, where the letters x and a label Alice’s measurement choice and outcome, respectively, each POVM having $o_{A}$ possible values.*

*(1) A state $\rho^{AB}$ of the system AB is said to be unsteerable from A to B with $M_{A}$ if there exists a probability distribution (PD) ${\left\{\pi_{\lambda }\right\}}_{\lambda =1}^{d}$ and a set of states ${\left\{\sigma_{\lambda }\right\}}_{\lambda =1}^{d}\subset D_{B}$ such that*

(1) $$\rho_{a|x}:={\mathrm{tr}}_{A}\left[\left(M_{a|x}⊗1_{B}\right)\rho^{AB}\right]=\sum_{\lambda =1}^{d}\pi_{\lambda }P_{A}\left(a|x,\lambda \right)\sigma_{\lambda },\;\forall x,a,$$

*where ${\left\{P_{A}\left(a|x,\lambda \right)\right\}}_{a=1}^{o_{A}}$ is a PD for each (x,λ). In this case, we also say that Equation (1) is an LHV-LHS model of $\rho^{AB}$ with respect to $M_{A}$.*

*(2) A state $\rho^{AB}$ is said to be steerable from A to B with $M_{A}$ if it is not unsteerable from A to B with $M_{A}$. In this case, we also say that $\rho^{AB}$ exhibits quantum steering with $M_{A}$.*

*(3) A state $\rho^{AB}$ is said to be unsteerable from A to B if for any $M_{A}$, $\rho^{AB}$ is unsteerable from A to B with $M_{A}$.*

*(4) A state $\rho^{AB}$ is said to be steerable from A to B if* ∃ *an $M_{A}$ such that it is steerable from A to B with $M_{A}$, i.e., it is not unsteerable from A to B with $M_{A}$.*

*Symmetrically, we define unsteerability and steerability of a state from B to A.*

*(5) A state $\rho^{AB}$ is said to be steerable if it is steerable from A to B or B to A.*

*(6) A state $\rho^{AB}$ is said to be unsteerable if it is not steerable, i.e., it is unsteerable both from A to B, and B to A.*

Here are some remarks to the definitions above.

**Remark** **1.** *Denote by ${\mathrm{US}}_{A}\left(M_{A}\right)$ the set of all states which are unsteerable from A to B with respect to $M_{A}$, by ${\mathrm{US}}_{A}$ the set of all states which are ussteerable from A to B, and denote by $S_{A}\left(M_{A}\right)$ the set of all states which are steerable from A to B with $M_{A}$, by $S_{A}$ the set of all states which are steerable from A to B. From the definition above, we have*

(2) $${\mathrm{US}}_{A}=\underset{M_{A}}{⋂}{\mathrm{US}}_{A}\left(M_{A}\right);S_{A}=\underset{M_{A}}{⋃}S_{A}\left(M_{A}\right).$$

**Remark** **2.** *The physical interpretation is as follows. When a state $\rho^{AB}$ is unsteerable with $M_{A}$, Bob can interpret his conditional states $\rho_{a|x}:={\mathrm{tr}}_{A}\left[\left(M_{a|x}⊗1_{B}\right)\rho^{AB}\right]$ by Equation (1) as coming from the pre-existing states ${\left\{\sigma_{\lambda }\right\}}_{\lambda =1}^{d}$ and the PD ${\left\{\pi_{\lambda }\right\}}_{\lambda =1}^{d}$, where only the probabilities are changed due to the knowledge ${\left\{P_{A}\left(a|x,\lambda \right)\right\}}_{x,a,\lambda }$ of Alice’s measurement choice x and outcome a.*

**Example** **1.** *Let us now assume that Alice’s measurements in $M_{A}$ are compatible, in the sense of being jointly measurable [29]. This means that there exists a single ‘parent’ POVM $N={\left\{N_{\lambda }\right\}}_{\lambda =1}^{d}$ such that $\forall M^{x}={\left\{M_{a|x}\right\}}_{a=1}^{o_{A}}\in M_{A}$, there is d PDs ${\left\{P_{A}\left(a|x,\lambda \right)\right\}}_{a=1}^{o_{A}}\left(\lambda =1,2,\ldots ,d\right)$, such that*

$$M_{a|x}=\sum_{\lambda =1}^{d}P_{A}\left(a|x,\lambda \right)N_{\lambda }\left(a=1,2,\ldots ,o_{A}\right).$$

*Thus, for any state $\rho^{AB}$ of the system AB, we have for each (a,x),*

$${\mathrm{tr}}_{A}\left[\left(M_{a|x}⊗1\right)\rho^{AB}\right]=\sum_{\lambda =1}^{d}P_{A}\left(a|x,\lambda \right){\mathrm{tr}}_{A}\left[\left(N_{\lambda }⊗1\right)\rho^{AB}\right]=\sum_{\lambda =1}^{d}\pi_{\lambda }P_{A}\left(a|x,\lambda \right)\sigma_{\lambda },$$

*where*

$$\pi_{\lambda }=\mathrm{tr}\left[\left(N_{\lambda }⊗1\right)\rho^{AB}\right],\;\sigma_{\lambda }=\frac{1}{\pi_{\lambda }}{\mathrm{tr}}_{A}\left[\left(N_{\lambda }⊗1\right)\rho^{AB}\right].$$

*This shows that every state $\rho^{AB}$ is unsteerable from A to B with a compatible measurement assemblage $M_{A}$.*

The following theorems were proved in [46].

**Theorem** **1.** *([46], Theorem 3.2) A state $\rho^{AB}$ of the system AB is unsteerable from A to B with $M_{A}$ if and only if there exists a PD ${\left\{\pi_{\lambda }\right\}}_{\lambda =1}^{d}$, a set of states ${\left\{\sigma_{\lambda }\right\}}_{\lambda =1}^{d}\subset D_{B}$, and $dm_{A}$ PDs ${\left\{P_{A}\left(a|x,\lambda \right)\right\}}_{a=1}^{o_{A}}\left(1\leq x\leq m_{A},1\leq \lambda \leq d\right)$ such that every local POVM ${\left\{N_{b}\right\}}_{b=1}^{o_{B}}$ of B, it holds that*

(3) $$\mathrm{tr}\left[\left(M_{a|x}⊗N_{b}\right)\rho^{AB}\right]=\sum_{\lambda =1}^{d}\pi_{\lambda }P_{A}\left(a|x,\lambda \right)\mathrm{tr}\left(N_{b}\sigma_{\lambda }\right),\;\;\forall x,a,b.$$

**Theorem** **2.** *([46], Theorem 3.3) A state $\rho^{AB}$ of the system AB is unsteerable from A to B if and only if for every $M_{A}$, there exists a PD ${\left\{\pi_{\lambda }\right\}}_{\lambda =1}^{d}$, a set of states ${\left\{\sigma_{\lambda }\right\}}_{\lambda =1}^{d}\subset D_{B}$ and $dm_{A}$ PDs ${\left\{P_{A}\left(a|x,\lambda \right)\right\}}_{a=1}^{o_{A}}\left(1\leq x\leq m_{A},1\leq \lambda \leq d\right)$ such that for every POVM ${\left\{N_{b}\right\}}_{b=1}^{o_{B}}$ of B, it holds that*

(4) $$\mathrm{tr}\left[\left(M_{a|x}⊗N_{b}\right)\rho^{AB}\right]=\sum_{\lambda =1}^{d}\pi_{\lambda }P_{A}\left(a|x,k\right)\mathrm{tr}\left(N_{b}\sigma_{\lambda }\right),\;\;\forall x,a,b,$$

## 3. EPR Steering Inequalities

Let $B_{\mathrm{her}}\left(H_{A}⊗H_{B}\right)$ be the set of all hermitian operators of the system $H_{A}⊗H_{B}$.

**Theorem** **3.** *Suppose that $A_{i}\in B_{\mathrm{her}}\left(H_{A}\right),B_{i}\in B_{\mathrm{her}}\left(H_{B}\right)\left(i=1,2,\ldots ,n\right)$ and there exists a positive constant M such that*

(5) $$\sum_{i=1}^{n}{\left|\mathrm{tr}\left(B_{i}T\right)\right|}^{2}\leq M,\;\;\forall T\in D_{B}.$$

*Then for every $\rho \in {\mathrm{US}}_{A}$, it holds that*

(6) $$F_{n}\left(\rho ,\mu \right):=\frac{1}{\sqrt{n}}\left|\sum_{i=1}^{n}{\langle A_{i}⊗B_{i}\rangle }_{\rho }\right|\leq \sqrt{\frac{M}{n}}\sqrt{\sum_{i=1}^{n}r{\left(A_{i}\right)}^{2}},$$

*where $\mu =\{A_{1},A_{2},\ldots ,A_{n};B_{1},B_{2},\ldots ,B_{n}\}$, $r(A_{i})$ is the spectral radius of $A_{i}$.*

**Proof.**  Since $A_{i}\in B_{\mathrm{her}}\left(H_{A}\right),B_{i}\in B_{\mathrm{her}}\left(H_{B}\right),i=1,2,\ldots ,n$, then the following spectrum decompositions are valid:

(7) $$A_{i}=\sum_{j=1}^{m_{1}}\lambda_{j}^{\left(i\right)}P_{j}^{\left(i\right)},\;B_{i}=\sum_{k=1}^{m_{2}}\mu_{k}^{\left(i\right)}Q_{k}^{\left(i\right)}\;\left(i=1,2,\ldots ,n\right).$$

Consider POVMs $M^{i}=\left\{P_{j}^{\left(i\right)},j=1,2,\ldots ,m_{1}\right\},\;N^{i}=\left\{Q_{k}^{\left(i\right)},k=1,2,\ldots ,m_{2}\right\}\left(i=1,2,\ldots ,n\right),$ and the measurement assemblages $M_{A}=\left\{M^{1},M^{2},\ldots ,M^{n}\right\},\;N_{B}=\left\{N^{1},N^{2},\ldots ,N^{n}\right\}$. Suppose that $\rho \in {\mathrm{US}}_{A}$, then $\rho \in {\mathrm{US}}_{A}\left(M_{A}\right)$. Thus, we see from Theorem 2 that there exists a PD ${\left\{\pi_{\lambda }\right\}}_{\lambda =1}^{d}$, a set of states ${\left\{\sigma_{\lambda }\right\}}_{\lambda =1}^{d}\subset D_{B}$, and nd PDs $\{P_{A}\left(j|x,\lambda \right),j=1,2,\ldots ,m_{1}\}\left(1\leq x\leq n,1\leq \lambda \leq d\right)$ such that

(8) $$\mathrm{tr}\left[\left(P_{j}^{\left(x\right)}⊗Q_{k}^{\left(y\right)}\right)\rho \right]=\sum_{\lambda =1}^{d}\pi_{\lambda }P_{A}\left(j|x,\lambda \right)\mathrm{tr}\left(Q_{k}^{\left(y\right)}\sigma_{\lambda }\right)$$

for all x,y=1,2,…,n and all $j\in \left\{1,2,\ldots ,m_{1}\right\},k\in \left\{1,2,\ldots ,m_{2}\right\}$. Hence, by Equations (7) and (8), we compute that

$$\begin{array}{lll} {\langle A_{i}⊗B_{i}\rangle }_{\rho } & = & \sum_{j=1}^{m_{1}}\sum_{k=1}^{m_{2}}\lambda_{j}^{\left(i\right)}\mu_{k}^{\left(i\right)}{\langle P_{j}^{\left(i\right)}⊗Q_{k}^{\left(i\right)}\rangle }_{\rho }\\ & = & \sum_{j=1}^{m_{1}}\sum_{k=1}^{m_{2}}\lambda_{j}^{\left(i\right)}\mu_{k}^{\left(i\right)}\sum_{\lambda =1}^{d}\pi_{\lambda }P_{A}\left(j|i,\lambda \right)\mathrm{tr}\left(Q_{k}^{\left(i\right)}\sigma_{\lambda }\right)\\ & = & \sum_{\lambda =1}^{d}\pi_{\lambda }L_{i}\left(\lambda \right), \end{array}$$

where

$$\begin{array}{lll} L_{i}\left(\lambda \right) & = & \left(\sum_{j=1}^{m_{1}}\lambda_{j}^{\left(i\right)}P_{A}\left(j|i,\lambda \right)\right)\left(\sum_{k=1}^{m_{2}}\mu_{k}^{\left(i\right)}\mathrm{tr}\left(Q_{k}^{\left(i\right)}\sigma_{\lambda }\right)\right)\\ & = & \left(\sum_{j=1}^{m_{1}}\lambda_{j}^{\left(i\right)}P_{A}\left(j|i,\lambda \right)\right)\mathrm{tr}\left(B_{i}\sigma_{\lambda }\right). \end{array}$$

Thus, by Cauchy inequality and Equation (5), we have

$$\begin{array}{ccc} \left|\sum_{i=1}^{n}{\langle A_{i}⊗B_{i}\rangle }_{\rho }\right| & = & \left|\sum_{i=1}^{n}\sum_{\lambda =1}^{d}\pi_{\lambda }L_{i}\left(\lambda \right)\right|\\ & \leq & \sum_{\lambda =1}^{d}\pi_{\lambda }\left|\sum_{i=1}^{n}L_{i}\left(\lambda \right)\right|\\ & = & \sum_{\lambda =1}^{d}\pi_{\lambda }\left|\sum_{i=1}^{n}\left(\sum_{j=1}^{m_{1}}\lambda_{j}^{\left(i\right)}P_{A}\left(j|i,\lambda \right)\right)\mathrm{tr}\left(B_{i}\sigma_{\lambda }\right)\right|\\ & \leq & \sum_{\lambda =1}^{d}\pi_{\lambda }\sqrt{\sum_{i=1}^{n}{\left(\sum_{j=1}^{m_{1}}\lambda_{j}^{\left(i\right)}P_{A}\left(j|i,\lambda \right)\right)}^{2}}\sqrt{\sum_{i=1}^{n}{\left(\mathrm{tr}(B_{i}\sigma_{\lambda })\right)}^{2}}\\ & \leq & \sum_{\lambda =1}^{d}\pi_{\lambda }\sqrt{\sum_{i=1}^{n}r{\left(A_{i}\right)}^{2}{\left(\sum_{j=1}^{m_{1}}P_{A}\left(j|i,\lambda \right)\right)}^{2}}\sqrt{M}\\ & \leq & \sqrt{M}\sqrt{\sum_{i=1}^{n}r{\left(A_{i}\right)}^{2}}. \end{array}$$

□

In Theorem 3, we see that if the inequality (6) is invalid for any observables $\left\{A_{i},B_{i}\right\}$ satisfying the condition there, then the state ρ must be steerable. Thus, the violating of the inequality implies the steerability of the state ρ. Since this, we call the inequality a *steering inequality*.

In particular, let $H_{A}=H_{B}=C^{2}$, and

(9) $$A_{i}={\vec{a}}_{i}\vec{\sigma }=a_{i}^{1}\sigma_{x}+a_{i}^{2}\sigma_{y}+a_{i}^{3}\sigma_{z},\;\;B_{i}={\vec{b}}_{i}\vec{\sigma }=b_{i}^{1}\sigma_{x}+b_{i}^{2}\sigma_{y}+b_{i}^{3}\sigma_{z},$$

where ${\vec{a}}_{i}={\left(a_{i}^{1},a_{i}^{2},a_{i}^{3}\right)}^{T}$ are unit vectors in $R^{3}$ for all i=1,2,…,n and ${\vec{b}}_{i}={\left(b_{i}^{1},b_{i}^{2},b_{i}^{3}\right)}^{T}\left(i=1,2,\ldots ,n\right)$ are orthonormal vectors in $R^{3}$. Then $A_{i},B_{i}$ are all self-adjoint unitary operators of trace 0 for all *i*, thus the eigenvalues of $A_{i}$ are all 1,−1, we get $r(A_{i})=1$.

Since ${\vec{b}}_{i}={\left(b_{i}^{1},b_{i}^{2},b_{i}^{3}\right)}^{T}\left(i=1,2,\ldots ,n\right)$ are orthonormal vectors, we can easily obtain that operators $\frac{1}{\sqrt{2}}I,\frac{1}{\sqrt{2}}B_{i},i=1,2,\ldots ,n$ are orthonormal. Thus, the Bessel inequality yields that

$$\frac{1}{2}\left({|\langle I,\eta \rangle }_{HS}{|}^{2}+\sum_{i=1}^{n}{\left|{\langle B_{i},\eta \rangle }_{HS}\right|}^{2}\right)\leq \mathrm{tr}\left(\eta^{2}\right)\leq 1,\;\;\forall \eta \in D_{B},$$

and so

(10) $$\sum_{i=1}^{n}|\mathrm{tr}\left(B_{i}\eta \right){|}^{2}=\sum_{i=1}^{n}{\left|{\langle B_{i},\eta \rangle }_{HS}\right|}^{2}\leq 1,\forall \eta \in D_{B}.$$

Thus, inequality (5) is valid for M=1 and then we obtain the following result which was pointed out in [43] without proof.

**Corollary** **1.** *Suppose that $A_{i},B_{i},i=1,2,\ldots ,n$ are given in Equation (9). Then*

(11) $$F_{n}\left(\rho ,\mu \right)=\frac{1}{\sqrt{n}}\left|\sum_{i=1}^{n}{\langle A_{i}⊗B_{i}\rangle }_{\rho }\right|\leq 1,\;\;\forall \rho \in {\mathrm{US}}_{A},$$

*where $\mu =\{{\vec{a}}_{1},{\vec{a}}_{2},\ldots ,{\vec{a}}_{n};{\vec{b}}_{1},{\vec{b}}_{2},\ldots ,{\vec{b}}_{n}\}.$*

**Example** **2.** *For the maximally entangled 2-qubit state $|\psi \rangle =\frac{1}{\sqrt{2}}(|00\rangle +\left|11\rangle \right)$, we have*

$$\rho =\left|\psi \rangle \langle \psi \right|=\frac{1}{2}(|00\rangle \langle 00|+\left|00\rangle \langle 11\right|+\left|11\rangle \langle 00\right|+|11\rangle \langle 11|).$$

*Generally, for all real unit vectors: $\vec{a}={\left(a_{x},a_{y},a_{z}\right)}^{T},\;\vec{b}={\left(b_{x},b_{y},b_{z}\right)}^{T},$ and the Pauli operator vector $\vec{\sigma }={\left(\sigma_{x},\sigma_{y},\sigma_{z}\right)}^{T},$ we have*

$$\begin{array}{c} \vec{a}\cdot \vec{\sigma }⊗\vec{b}\cdot \vec{\sigma }=\left(a_{x}\sigma_{x}+a_{y}\sigma_{y}+a_{z}\sigma_{z}\right)⊗\left(b_{x}\sigma_{x}+b_{y}\sigma_{y}+b_{z}\sigma_{z}\right)=\sum_{i,j}a_{i}b_{j}\sigma_{i}⊗\sigma_{j}, \end{array}$$

*with ${\langle \sigma_{i}⊗\sigma_{j}\rangle }_{\rho }=0$ for all i,j∈{x,y,z} except for the following three cases:*

$${\langle \sigma_{x}⊗\sigma_{x}\rangle }_{\rho }=1,{\langle \sigma_{y}⊗\sigma_{y}\rangle }_{\rho }=-1,{\langle \sigma_{z}⊗\sigma_{z}\rangle }_{\rho }=1.$$

*Thus,*

(12) $${\langle \vec{a}\cdot \vec{\sigma }⊗\vec{b}\cdot \vec{\sigma }\rangle }_{\rho }=a_{x}b_{x}-a_{y}b_{y}+a_{z}b_{z}.$$

*In particular, put n=3, and*

(13) $$A_{1}=\sigma_{x},B_{1}=\frac{\sqrt{3}}{2}\sigma_{x}+\frac{1}{2}\sigma_{y},A_{2}=\sigma_{y},B_{2}=\frac{1}{2}\sigma_{x}-\frac{\sqrt{3}}{2}\sigma_{y},A_{3}=\sigma_{z},B_{3}=\sigma_{z},$$

*we obtain*

$$\begin{array}{c} F_{3}\left(\rho ,\mu \right)=\frac{1}{\sqrt{3}}\left|\sum_{i=1}^{3}{\langle A_{i}⊗B_{i}\rangle }_{\rho }\right|=\frac{\sqrt{3}+1}{\sqrt{3}}>1. \end{array}$$

*By Corollary 1, we get that ρ=|ψ〉〈ψ| is steerable from A to B.*

**Example** **3.** *The 2-qubit state ρ=|ψ〉〈ψ| is steerable from A to B, where $|\psi \rangle =r_{0}|00\rangle +r_{1}\left|11\rangle ,\right|r_{0}r_{1}|>\frac{\sqrt{3}-1}{4},r_{0},r_{1}\in R$.*

*By computation, we obtain*

$$\rho =\left|\psi \rangle \langle \psi \right|=r_{0}^{2}\left|00\rangle \langle 00\right|+r_{0}r_{1}\left|00\rangle \langle 11\right|+r_{0}r_{1}\left|11\rangle \langle 00\right|+r_{1}^{2}|11\rangle \langle 11|.$$

*Generally, for all real unit vectors: $\vec{a}={\left(a_{x},a_{y},a_{z}\right)}^{T},\;\vec{b}={\left(b_{x},b_{y},b_{z}\right)}^{T},$ and the Pauli operator vector $\vec{\sigma }={\left(\sigma_{x},\sigma_{y},\sigma_{z}\right)}^{T},$ we obtain*

$$\begin{array}{c} \vec{a}\cdot \vec{\sigma }⊗\vec{b}\cdot \vec{\sigma }=\sum_{i,j}a_{i}b_{j}\sigma_{i}⊗\sigma_{j}, \end{array}$$

*with ${\langle \sigma_{i}⊗\sigma_{j}\rangle }_{\rho }=0$ for all i,j∈{x,y,z} except for the following four cases*

$${\langle \sigma_{x}⊗\sigma_{x}\rangle }_{\rho }=2r_{0}r_{1},{\langle \sigma_{y}⊗\sigma_{y}\rangle }_{\rho }=-2r_{0}r_{1},{\langle \sigma_{z}⊗\sigma_{z}\rangle }_{\rho }=1.$$

*Thus,*

$${\langle \vec{a}\cdot \vec{\sigma }⊗\vec{b}\cdot \vec{\sigma }\rangle }_{\rho }=2r_{0}r_{1}a_{x}b_{x}-2r_{0}r_{1}a_{y}b_{y}+a_{z}b_{z}.$$

*Particularly, take n=3, and*

$$A_{1}=\sigma_{x},\;A_{2}=\sigma_{y},\;A_{3}=\sigma_{z};$$

$$B_{1}=\frac{m}{4r_{0}r_{1}}\sigma_{x}+\sqrt{1-{\left(\frac{m}{4r_{0}r_{1}}\right)}^{2}}\sigma_{y},\;B_{2}=\sqrt{1-{\left(\frac{m}{4r_{0}r_{1}}\right)}^{2}}\sigma_{x}-\frac{m}{4r_{0}r_{1}}\sigma_{y},\;B_{3}=\sigma_{z},$$

*where $\sqrt{3}-1<m<4\left|r_{0}r_{1}\right|$, we get*

$$\begin{array}{c} F_{3}\left(\rho ,\mu \right)=\frac{1}{\sqrt{3}}\left|\sum_{i=1}^{3}{\langle A_{i}⊗B_{i}\rangle }_{\rho }\right|=\frac{m+1}{\sqrt{3}}>1. \end{array}$$

*By Corollary 1, we get that ρ=|ψ〉〈ψ| is steerable from A to B.*

Any two-qubit state can be written in the following form

(14) $$\rho =\frac{1}{4}\left(I⊗I+\vec{a}\cdot \vec{\sigma }⊗I+I⊗\vec{b}\cdot \vec{\sigma }+\sum_{i,j=1}^{3}t_{ij}\sigma_{i}⊗\sigma_{j}\right),$$

where $\sigma_{j},j=1,2,3$ are three Pauli matrices, $\vec{\sigma }={\left(\sigma_{1},\sigma_{2},\sigma_{3}\right)}^{T}$ is the vector composed of these Pauli matrices, $T_{\rho }=\left[t_{ij}\right]$ is the correlation matrix of ρ, $T_{\rho }^{†}T_{\rho }$ with eigenvalues $\lambda_{1}\left(\rho \right)\geq \lambda_{2}\left(\rho \right)\geq \lambda_{3}\left(\rho \right)$.

As an application of Corollary 1, we have the following result.

**Corollary** **2.** *Let ${\vec{a}}_{i},{\vec{b}}_{i},i=1,2,\ldots ,n$ be as in Equation (9) and $M_{A}=\left\{\left\{\frac{I+{\vec{a}}_{i}\cdot \vec{\sigma }}{2},\frac{I-{\vec{a}}_{i}\cdot \vec{\sigma }}{2}\right\}:i=1,2,\ldots ,n\right\}$. If $\rho \in {\mathrm{US}}_{A}\left(M_{A}\right)$, then it holds that*

(15) $$\frac{1}{\sqrt{n}}\left|\sum_{i=1}^{n}\langle {\vec{a}}_{i},T_{\rho }{\vec{b}}_{i}\rangle \right|\leq 1.$$

**Proof.**  Let $\rho \in {\mathrm{US}}_{A}\left(M_{A}\right)$. Then, we see from Corollary 1 that

$$\begin{array}{ccc} 1 & \geq & \frac{1}{\sqrt{n}}\left|\sum_{i=1}^{n}{\langle A_{i}⊗B_{i}\rangle }_{\rho }\right|\\ & = & \frac{1}{\sqrt{n}}\left|\sum_{i=1}^{n}{\langle {\vec{a}}_{i}\cdot \vec{\sigma }⊗{\vec{b}}_{i}\cdot \vec{\sigma }\rangle }_{\rho }\right|\\ & = & \frac{1}{\sqrt{n}}\left|\sum_{i=1}^{n}\mathrm{tr}\left(({\vec{a}}_{i}\cdot \vec{\sigma }⊗{\vec{b}}_{i}\cdot \vec{\sigma })\rho \right)\right|\\ & = & \frac{1}{\sqrt{n}}\left|\sum_{i=1}^{n}\sum_{k,j=1}^{3}a_{i}^{k}t_{kj}b_{i}^{j}\right|\\ & = & \frac{1}{\sqrt{n}}\left|\sum_{i=1}^{n}\langle {\vec{a}}_{i},T_{\rho }{\vec{b}}_{i}\rangle \right|. \end{array}$$

□

It was proved in ([25], Theorem 2) that a Bell diagonal state ρ is steerable with three projective measurements if $∥T_{\rho }{∥}_{F}^{2}=\lambda_{1}\left(\rho \right)+\lambda_{2}\left(\rho \right)+\lambda_{3}\left(\rho \right)>1$. We see from Corollary 2 that if the inequality (15) is not valid, then the state ρ must be steerable with *n* projective measurements $\left\{\frac{I+{\vec{a}}_{i}\cdot \vec{\sigma }}{2},\frac{I-{\vec{a}}_{i}\cdot \vec{\sigma }}{2}\right\}$ (i=1,2,…,n). For instance, we have Corollary 3 and Corollary 4 below, which give sufficient conditions for a general two-qubit state to be steerable under two and three projective measurements, respectively. In [26], a strong necessary condition was obtained for the steerability of two-qubit states having maximally mixed reduced states, via the construction of local hidden state models and two provably sufficient conditions were also obtained, via asymmetric EPR steering inequalities.

**Corollary** **3.** *Suppose that $\rho \in D(C^{2}⊗C^{2})$ with $\lambda_{i}\left(\rho \right)>0\left(i=1,2\right)$ and $\sqrt{\lambda_{1}\left(\rho \right)}+\sqrt{\lambda_{2}\left(\rho \right)}>\sqrt{2}$, then $\rho \in S_{A}\left(M_{A}\right)$, where*

$$M_{A}=\left\{\left\{\frac{I+{\vec{a}}_{i}\cdot \vec{\sigma }}{2},\frac{I-{\vec{a}}_{i}\cdot \vec{\sigma }}{2}\right\}:i=1,2\right\},{\vec{a}}_{1}=\frac{T_{\rho }{\vec{b}}_{1}}{|T_{\rho }{\vec{b}}_{1}|},\;{\vec{a}}_{2}=\frac{T_{\rho }{\vec{b}}_{2}}{|T_{\rho }{\vec{b}}_{2}|},$$

*and ${\vec{b}}_{1},{\vec{b}}_{2}$ are the orthonormal eigenvectors corresponding to the first two largest eigenvalues $\lambda_{1}\left(\rho \right),\lambda_{2}\left(\rho \right)$ of $T_{\rho }^{†}T_{\rho }$, respectively.*

**Proof.**  We compute that $|T_{\rho }{\vec{b}}_{i}|=\sqrt{\lambda_{i}\left(\rho \right)}\left(i=1,2\right)$ and so

$$\frac{1}{\sqrt{2}}\left|\sum_{i=1}^{2}\langle {\vec{a}}_{i},T_{\rho }{\vec{b}}_{i}\rangle \right|=\frac{1}{\sqrt{2}}\sum_{i=1}^{2}\sqrt{\lambda_{i}\left(\rho \right)}>1.$$

Thus, Corollary 2 yields that $\rho \in S_{A}\left(M_{A}\right)$. □

Similarly, we can arrive the following conclusion for the case of n=3.

**Corollary** **4.** *Suppose that $\rho \in D(C^{2}⊗C^{2})$ with $\lambda_{i}\left(\rho \right)>0\left(i=1,2,3\right)$ and $\sum_{i=1}^{3}\sqrt{\lambda_{i}\left(\rho \right)}>\sqrt{3}$, then $\rho \in S_{A}\left(M_{A}\right)$, where*

$$M_{A}=\left\{\left\{\frac{I+{\vec{a}}_{i}\cdot \vec{\sigma }}{2},\frac{I-{\vec{a}}_{i}\cdot \vec{\sigma }}{2}\right\}:i=1,2,3\right\},{\vec{a}}_{1}=\frac{T_{\rho }{\vec{b}}_{1}}{|T_{\rho }{\vec{b}}_{1}|},\;{\vec{a}}_{2}=\frac{T_{\rho }{\vec{b}}_{2}}{|T_{\rho }{\vec{b}}_{2}|},\;{\vec{a}}_{3}=\frac{T_{\rho }{\vec{b}}_{3}}{|T_{\rho }{\vec{b}}_{3}|},$$

*and ${\vec{b}}_{1},{\vec{b}}_{2},{\vec{b}}_{3}$ are the orthonormal eigenvectors corresponding to the eigenvalues $\lambda_{1}\left(\rho \right),$$\lambda_{2}\left(\rho \right),$$\lambda_{3}\left(\rho \right)$ of $T_{\rho }^{†}T_{\rho }$, respectively.*

**Proof.**  We compute that $|T_{\rho }{\vec{b}}_{i}|=\sqrt{\lambda_{i}\left(\rho \right)}\left(i=1,2,3\right)$ and so

$$\frac{1}{\sqrt{3}}\left|\sum_{i=1}^{3}\langle {\vec{a}}_{i},T_{\rho }{\vec{b}}_{i}\rangle \right|=\frac{1}{\sqrt{3}}\sum_{i=1}^{3}\sqrt{\lambda_{i}\left(\rho \right)}>1.$$

Thus, Corollary 2 yields $\rho \in S_{A}\left(M_{A}\right)$. □

**Example** **4.** *Consider the state ρ characterized by the correlation matrix*

$$T_{\rho }=\left(\begin{array}{ccc} t_{1} & 0 & 0\\ 0 & t_{2} & 0\\ 0 & 0 & t_{3} \end{array}\right)$$

*where $t_{i}\neq 0\left(i=1,2,3\right)$ and $|t_{1}\left|+\right|t_{2}\left|+\right|t_{3}|>\sqrt{3}.$ We note that $\lambda_{i}\left(\rho \right)>0\left(i=1,2,3\right)$ and*

$$\sqrt{\lambda_{1}\left(\rho \right)}+\sqrt{\lambda_{2}\left(\rho \right)}+\sqrt{\lambda_{3}\left(\rho \right)}=|t_{1}\left|+\right|t_{2}\left|+\right|t_{3}|>\sqrt{3}.$$

*Hence, we get from Corollary 4 that $\rho \in S_{A}\left(M_{A}\right)$, where*

(16) $$M_{A}=\left\{\left\{\frac{I+{\vec{a}}_{i}\cdot \vec{\sigma }}{2},\frac{I-{\vec{a}}_{i}\cdot \vec{\sigma }}{2}\right\}:i=1,2,3\right\}$$

*and ${\vec{a}}_{1}={\left(1,0,0\right)}^{T},{\vec{a}}_{2}={\left(0,1,0\right)}^{T},{\vec{a}}_{3}={\left(0,0,1\right)}^{T}$. In particular, the Bell state $\rho =|\beta_{10}\rangle \langle \beta_{10}|$ characterized by the correlation matrix*

$$T_{\rho }=\left(\begin{array}{ccc} -1 & 0 & 0\\ 0 & 1 & 0\\ 0 & 0 & 1 \end{array}\right),$$

*is steerable from A to B with $M_{A}$, where $|\beta_{10}\rangle =\frac{1}{\sqrt{2}}(|00\rangle -\left|11\rangle \right)$, $M_{A}$ is given in Equation (16).*

The following corollary gives a sufficient condition for a general two-qubit state to be steerable in terms of eigenvalues $\mu_{1},\mu_{2},\mu_{3}$ of $T_{\rho }$.

**Corollary** **5.** *Suppose that $\rho \in D(C^{2}⊗C^{2})$, and $T_{\rho }^{†}=T_{\rho }$, $\mu_{1},\mu_{2},\mu_{3}$ are the eigenvalues of $T_{\rho }$, ${\vec{b}}_{1},{\vec{b}}_{2},\;{\vec{b}}_{3}$ are the orthonormal eigenvectors corresponding to the eigenvalues $\mu_{1},\mu_{2},\mu_{3}$. Then*

*(a) When $|\mu_{1}+\mu_{2}|>\sqrt{2}$, $\rho \in S_{A}\left(M_{A}\right)$ where*

$$M_{A}=\left\{\left\{\frac{I+{\vec{a}}_{i}\cdot \vec{\sigma }}{2},\frac{I-{\vec{a}}_{i}\cdot \vec{\sigma }}{2}\right\}:i=1,2\right\},\;{\vec{a}}_{1}={\vec{b}}_{1},\;{\vec{a}}_{2}={\vec{b}}_{2}.$$

*(b) When $|\mu_{1}+\mu_{2}+\mu_{3}|>\sqrt{3}$, $\rho \in S_{A}\left(M_{A}\right)$ where*

$$M_{A}=\left\{\left\{\frac{I+{\vec{a}}_{i}\cdot \vec{\sigma }}{2},\frac{I-{\vec{a}}_{i}\cdot \vec{\sigma }}{2}\right\}:i=1,2,3\right\},\;{\vec{a}}_{1}={\vec{b}}_{1},\;{\vec{a}}_{2}={\vec{b}}_{2},\;{\vec{a}}_{3}={\vec{b}}_{3}.$$

**Proof.**  (a) Let $|\mu_{1}+\mu_{2}|>\sqrt{2}$. Since ${\vec{a}}_{1}={\vec{b}}_{1},\;{\vec{a}}_{2}={\vec{b}}_{2}$, and ${\vec{b}}_{1},{\vec{b}}_{2}$ are the orthonormal eigenvectors corresponding to the eigenvalues $\mu_{1},\mu_{2}$ of $T_{\rho }$, respectively, we have

$$\frac{1}{\sqrt{2}}\left|\sum_{i=1}^{2}\langle {\vec{a}}_{i},T_{\rho }{\vec{b}}_{i}\rangle \right|=\frac{1}{\sqrt{2}}\left|\sum_{i=1}^{2}\mu_{i}\langle {\vec{a}}_{i},{\vec{b}}_{i}\rangle \right|=\frac{1}{\sqrt{2}}\left|\sum_{i=1}^{2}\mu_{i}\right|>1,$$

since $|\mu_{1}+\mu_{2}|>\sqrt{2}$. It follows from Corollary 2 that $\rho \in S_{A}\left(M_{A}\right)$.

(b) Let $|\mu_{1}+\mu_{2}+\mu_{3}|>\sqrt{3}$. Since ${\vec{b}}_{1},{\vec{b}}_{2},{\vec{b}}_{3}$ are the orthonormal eigenvectors corresponding to the eigenvalues $\mu_{1},\mu_{2},\mu_{3}$ of $T_{\rho }$, respectively, and ${\vec{a}}_{1}={\vec{b}}_{1},\;{\vec{a}}_{2}={\vec{b}}_{2},\;{\vec{a}}_{3}={\vec{b}}_{3}$, we compute

$$\frac{1}{\sqrt{3}}\left|\sum_{i=1}^{3}\langle {\vec{a}}_{i},T_{\rho }{\vec{b}}_{i}\rangle \right|=\frac{1}{\sqrt{3}}\left|\sum_{i=1}^{3}\mu_{i}\langle {\vec{a}}_{i},{\vec{b}}_{i}\rangle \right|=\frac{1}{\sqrt{3}}\left|\sum_{i=1}^{3}\mu_{i}\right|>1,$$

since $|\mu_{1}+\mu_{2}+\mu_{3}|>\sqrt{3}$. It follows from Corollary 2 that $\rho \in S_{A}\left(M_{A}\right)$. □

**Example** **5.** *Consider the steerability of an "X" state given in [48]*

$$\begin{array}{c} \rho_{X}=\left(\begin{array}{cccc} v_{1} & & & v_{5}\\ & v_{2} & v_{6} & \\ & v_{6} & v_{3} & \\ v_{5} & & & v_{4} \end{array}\right) \end{array}$$

*where $v_{k}$’s are real parameters satisfying $v_{1}+v_{2}+v_{3}+v_{4}=1,v_{5}^{2}\leq v_{1}v_{4},v_{6}^{2}\leq v_{2}v_{3}$. It is not necessarily a Bell-diagonal state.*

*By computation, we get that the correlation matrix*

$$T_{\rho_{X}}=\left(\begin{array}{ccc} 2v_{5}+2v_{6} & 0 & 0\\ 0 & 2v_{6}-2v_{5} & 0\\ 0 & 0 & v_{1}-v_{2}-v_{3}+v_{4} \end{array}\right).$$

*We can easily see that the eigenvalues of $T_{\rho_{X}}$ are $2v_{5}+2v_{6},2v_{6}-2v_{5},v_{1}-v_{2}-v_{3}+v_{4}$ with the corresponding eigenstates ${\vec{a}}_{1}={\left(1,0,0\right)}^{T},{\vec{a}}_{2}={\left(0,1,0\right)}^{T},{\vec{a}}_{3}={\left(0,0,1\right)}^{T}$. Put*

$$M_{i}=\left\{\frac{I+{\vec{a}}_{i}\cdot \vec{\sigma }}{2},\frac{I-{\vec{a}}_{i}\cdot \vec{\sigma }}{2}\right\},$$

*then $M_{i}$ is a POVM for i=1,2,3. The steerability of $\rho_{X}$ is as follows.*

*(a) When $|v_{6}|>\frac{\sqrt{2}}{4}$ or $|v_{1}-v_{2}-v_{3}+v_{4}+2v_{5}+2v_{6}|>\sqrt{2}$ or $|v_{1}-v_{2}-v_{3}+v_{4}+2v_{6}-2v_{5}|>\sqrt{2}$, the condition $|\mu_{1}+\mu_{2}|>\sqrt{2}$ in Corollary 5 is satisfied and so $\rho_{X}\in S_{A}\left(M_{A}\right)$ where $M_{A}=\left\{M_{1},M_{2}\right\}$.*

*(b) When $|v_{1}-v_{2}-v_{3}+v_{4}+4v_{6}|>\sqrt{3}$, the condition $|\mu_{1}+\mu_{2}+\mu_{3}|>\sqrt{3}$ in Corollary 5 is satisfied and so $\rho_{X}\in S_{A}\left(M_{A}\right)$ where $M_{A}=\left\{M_{1},M_{2},M_{3}\right\}$.*

## 4. Conclusions

In this paper, we have obtained some remarks on EPR steering of bipartite states, including mathematical definitions and characterizations of steerability. Using the characterizations, we have established some necessary conditions for a state to be unsteerable by proving some inequalities. The validity of the derived inequalities are necessary for unsteerability of bipartite states, and then the violation of some of them are sufficient for a state to be steerable. As applications, the EPR steerability of the maximally entangled states is checked and some conditions for the steerability of the X-states are obtained.
